# Persistence and gender differences in protection against severe fever with thrombocytopaenia syndrome virus with natural infection: a 4-year follow-up and mathematical prediction study

**DOI:** 10.1017/S1469440918003643

**Published:** 2019-01-30

**Authors:** R. Qi, Y.T. Huang, X.J. Yu

**Affiliations:** 1 Wuhan University School of Health Sciences, Wuhan, China; 2 State Key Laboratory of Virology, Wuhan University, Wuhan, China; 3 Shandong University, Jinan, Shandong Province, China

**Keywords:** Bunyaviruses, emerging infections, epidemiology, SFTS virus

## Abstract

Severe fever with thrombocytopaenia syndrome (SFTS) is an emerging infectious disease discovered in 2010 and has a case fatality as high as 30%. We intended to study the immune protection conferred by SFTS with natural infection. We collected and analysed 4-year follow-up data to study the characteristics of neutralising antibodies against SFTS virus (SFTSV). The 50% plaque reduction neutralisation test was used for the detection of neutralising antibodies against SFTSV. Geometric mean titres (GMTs) and proportions of patients with a protective titre were analysed, and the persistence of protection was predicted. The titre of antibodies declined yearly in the 4-year study period. Approximately 3 months after infection, the GMT was 143 (95% confidence interval (CI): 89–231), and 100% of patients had a protective titre. In the fourth year, the GMT declined to 53 (95% CI: 37–76), and 95% of patients had a protective titre. The titre was higher in females than in males. On average, the protection offered by neutralising antibodies against SFTSV could last as long as 9 years. The durations of protection were different for different initial titres. The characteristics of neutralising antibodies can be used as a reference for the vaccination doses and schedules of forthcoming vaccines.

## Introduction

Severe fever with thrombocytopaenia syndrome (SFTS) is an emerging infectious disease discovered in China in 2010 [1]. SFTS occurs in rural areas, targeting people >50 years old [1–3]. While the case fatality rate is approximately 6.4% nationwide in China, the initial case fatality rate is as high as 30% [1, 3]. Unfortunately, there is no curative treatment for SFTS. A safe and efficacious vaccine may be a good option. However, there are no vaccines available on the market right now. Information about the characteristics of immunity to SFTS is still scarce as it is a newly discovered disease. We conducted a follow-up study from 2011 to 2015 to study the decay of neutralising antibodies against SFTS virus (SFTSV). The titres and duration of neutralising antibodies can be used as guidance for the vaccination doses and schedules of forthcoming vaccines. After the study period of 4 years, all 25 patients still maintained neutralising antibodies, which indicated long-term persistence of neutralising antibodies against SFTSV. We further analysed the 4-year follow-up data to learn about the long-term persistence and the differences of neutralising antibodies against SFTSV between the gender and age of patients. We used mathematical methods to get a prediction based on this 4-year data. The generalised estimating equation (GEE) is a general statistical approach to fit a marginal model for longitudinal data analysis, and it has been popularly applied to clinical trials and biomedical studies [4, 5].

## Methods

### Data

The 4-year 50% plaque reduction neutralisation test (PRNT_50_) data were obtained from the detection of neutralising antibodies against SFTSV. The living patients were laboratory-confirmed by reverse transcription polymerase chain reaction (RT-PCR) to have SFTS, aged 42–75 years (median age 62 years), and from a rural area in Yiyuan County, Shandong Province, China. Blood samples were obtained from these patients three times from 2011 to 2015. The neutralising antibody titre against SFTSV was measured by standard plaque reduction neutralisation test. Serial twofold dilutions of sera samples were mixed with equal volumes of solution containing SFTSV for plaque formation. Plaques were counted, and the antibody PRNT_50_ titre was determined as the reciprocal of the highest serum dilution that reduced the SFTSV plaque count by 50% relative to the average number of plaques in viral control wells [6].

To date, there are still no reference criteria as to which PRNT_50_ titre could be defined as a protection threshold for SFTSV. Despite this, we took PRNT_50_ = 1:10 and PRNT_50_ = 1:20 as endpoints for predicting the duration of protection from neutralising antibody. In some articles studying neutralising antibodies against other viruses (e.g. Hantaan, Rift Valley fever, chikungunya, Japanese encephalitis), PRNT_50_ = 1:20 or PRNT_50_ = 1:10 was used as a negative cut-off [7–10]. Therefore, PRNT_50_ values of 10 or 20 were regarded as the surrogate endpoint. The predicted duration of neutralising antibodies indicated the time needed to decrease to these two titres.

### Statistical analysis

Two of the patients had higher titres in the fourth year than the first year. The reason was unknown; data from these two patients were excluded as ‘outliers’.

The geometric mean titres (GMT) along with their 95% confidence interval (CI) were calculated each year, stratified by gender and age. Ages were stratified into three groups (<60, 60–70 and >70 years old). We also calculated the proportion of patients with PRNT_50_ titre >1:20 or >1:10 with 95% CI. The Wilson method was used for CIs of proportions. This interval had good properties even for a small number or an extreme probability [11]. GMT and proportion were calculated based on non-missing values.

The declining trend of neutralising antibodies according to time was calculated using a linear model with the log_2_(PRNT_50_) titre as the response variable. The data of time variable in GEE models were not the numbers of visit, but rather the time from onset of disease to timing of visits. Models with additional group variables (gender, age, initial titre) were also performed. Multivariable regression analysis was conducted to explore real factors associated with duration of SFTSV antibodies. In multivariable regression, we included gender and age but not with initial titre. Initial titre was a patient's feature used to show how strongly the response of immunity to infection at first and could be affected by individual difference such as age and sex. The GEE was used to estimate the parameters of linear models with ‘exchangeable’ correlations. With an ‘exchangeable’ correlation, the model had minimum quasi-likelihood information criterion (QIC), which is a modification of Akaike's information criterion based on the ‘quasi-likelihood’ to select a working correlation structure in GEE models [12]. After the models were fitted, inverse predictions by the delta method were made to predict the time and 95% CI when the response variable values were specified. All analyses were run in R 3.3.1 with packages: binom (for CI of proportion), geepack (for GEE) and msm (for delta method) [13–16].

The study was approved by the ethics committee of Wuhan University. Informed consent was obtained from all participants.

## Results

Twenty-three of the 25 patients were analysed; two patients who showed up-trends were excluded as ‘outliers’. Because of varied disease onset for each patient, the time intervals between visits and onset were different. The interval between disease onset and the first visit was 3.6 months, on average, and ranged from 1.4 to 6.7 months. For the second visit, it was 1.7 years, on average, and ranged from approximately 1–2 years. For the third visit, it was 3.6 years, on average, and ranged from 2.8 to 3.9 years. Of all patients, 11 were male, and 12 were female. The median age for the 23 patients was 62 years old and ranged from 42 to 75 years old. There were ten patients aged <60, six aged 60−70 and seven aged >70 years old.

At the first visit, the GMT was 143 (95% CI: 89–231), and at the second visit, 1.7 years after disease onset, the GMT had declined to 69 (95% CI: 49–97). In the fourth year, the GMT had declined to 53 (95% CI: 37–76) (Table 1). The GMT was declining yearly, both in males and females, but there was a difference in titre at the first visit. The initial GMT for males was 104 (95% CI: 52–207), and the initial GMT for females was 181 (95% CI: 95–348). In all three age groups, GMTs were declining yearly. GMTs were similar in the <60-year-old group and 60–70-year-old group, but the GMT in >70-year-old group was smaller. GMTs with 95% CI by groups at each visit are shown in Table 2.

**Table 1.** GMTs and proportions of patients with titre >1:20 and 1:10 at three visits

Visit	Mean years after onset (range)	GMT (95% CI)^a^	Rate with titre >1:20 (95% CI) (%)	Rate with titre >1:10 (95% CI) (%)
First	0.3 (0.1–0.6)	143 (89–231)	89.47 (68.7–97.1)	100 (83.2–100)
Second	1.7 (0.9–2.1)	69 (49–97)	86.96 (67.9–95.5)	100 (85.7–100)
Third	3.6 (2.8–3.9)	53 (37–76)	80 (58.4–92.0)	95 (76.4–99.1)

a GMT: geometric mean titre, 95% CI: 95% confidence interval.

**Table 2.** GMTs and 95% CI by gender and age groups at three visits

Visit	Male (*n* = 11)	Female (*n* = 12)	<60 (*n* = 10)	60–70 (*n* = 6)	>70 (*n* = 7)
First	104 (52–207)	181 (95–348)	184 (97–346)	160 (53–485)	90 (33–243)
Second	48 (31–76)	95 (61–149)	70 (40–123)	80 (56–114)	59 (27–129)
Third	40 (27–59)	70 (39–124)	57 (41–78)	71 (29–173)	36 (17–76)

At the first visit, 89.5% of patients had PRNT_50_ > 1:20 and 100% of patients had PRNT_50_ > 1:10 (95% CI: 68.6–97% and 83.2–100%, respectively). At the second visit, 87% of patients had PRNT_50_ > 1:20 and 100% of patients still had PRNT_50_ > 1:10 (95% CI: 67.9–95.5% and 85.7–100%, respectively). At the last visit, the proportion of PRNT_50_ > 1:20 was 80% (95% CI: 58.4–92%). In contrast to the 100% PRNT_50_ > 1:10 seen at two previous visits, one patient had a PRNT_50_ = 1:10, 3.9 years after disease onset (Table 1).

The GEE was applied to estimate the persistence of neutralising antibodies. Log_2_(titre) declined significantly according to time (*P* < 0.001). On average, the time for antibodies to decline to 1:20 was estimated to be 6.7 years (95% CI: 5.1–8.3). If protection was defined as a PRNT_50_ titre >1:10, the duration of protection was estimated to be 9.2 years (95% CI: 7–11.4). Next, gender and age variables were added into the models, and as a result, dependent patterns of log_2_(titre) by time were significantly different between males and females (*P* = 0.050), but there was no significant difference among the age groups (aged 60–70 and aged >70 compared to aged <60: *P* = 0.96 and *P* = 0.49, respectively). The persistence of antibodies was predicted by gender. For males, the estimated duration of PRNT_50_ > 1:20 was 5.7 years (95% CI: 4.2–7.2), and for PRNT_50_ > 1:10, it was 8.2 years (95% CI: 6.2–10.2). For females, it took a longer time for PRNT_50_ to decline to 1:20 and 1:10. The duration was 7.9 and 10.4 years, respectively (95% CI: 6.4–9.4 and 8.4–12.5, respectively). Considering that the persistence of antibodies was relevant to the initial titre, we added initial titres as a group variable to the model and estimated the persistence by different initial titres. When the initial PRNT_50_ titre was 640, the duration, on average, was 11.6 years (1:20) (95% CI: 9.1–14.1) and 14.2 years (1:10) (95% CI: 10.7–17.8). When the initial titre was 320, the duration declined to 8 years (95% CI: 5.5–10.5) and 10.6 years (95% CI: 7.1–14.2) for PRNT_50_ 1:20 and 1:10, respectively. When the initial titre was 160, the estimated duration was 7.2 years (95% CI: 4.7–9.7) and 9.9 years (95% CI: 6.3–13.4) for PRNT_50_ 1:20 and 1:10, respectively. If the initial titre was <100 (80, 40, 20), it only took an average of 4 years (95% CI: 1.5–6.5) to decline to PRNT_50_ 1:20 and 6.6 years (95% CI: 3.1–10.1) to PRNT_50_ 1:10. An overview on the predicted duration of neutralising antibodies is summarised in Table 3.

**Table 3.** Estimated durations of titre declining to 1:10 and 1:20

	Declining to 1:10		Declining to 1:20	
	Duration (years)	95% CI^a^	Duration (years)	95% CI
All patients	9.2	6.9–11.4	6.7	5.1–8.3
Initial titre after infection				
640 (*n*^b^ = 4)	14.2	10.7–17.8	11.6	9.1–14.1
320 (*n* = 3)	10.6	7.1–14.2	8	5.5–10.5
160 (*n* = 5)	9.9	6.3–13.4	7.2	4.7–9.7
<100 (*n* = 7)^c^	6.6	3.1–10.1	4	1.5–6.5
Gender				
Female (*n* = 12)	10.4	8.4–12.5	7.9	6.4–9.4
Male (*n* = 11)	8.2	6.2–10.2	5.7	4.2–7.2

a CI: confidence interval.

b The number of patients.

c Initial titre <100 (*n* = 7) included two patients with titre = 80, three with titre = 40 and two with titre = 20.

## Discussion

Studies on the characteristics of neutralising antibodies against SFTSV have been scarce. A study [17] reported that neutralising antibody targeted to the SFTSV glycoprotein played a role in inhibiting SFTSV Gn/Gc-driven host cell entry. A similar result was seen in another study [18], which reported a monoclonal antibody neutralising SFTSV through blockage of the interactions between the Gn protein and the cellular receptor and indicated that the neutralising mechanism was mainly the inhibition of virus-cell attachment. Our study focused on the long-term immune response after infection with SFTSV. All the patients followed were farmers aged 42–75 years old. A study [3] based on national surveillance data of more than 5300 confirmed cases reported that 91.57% of the reported cases were older than 40 years of age (40–80 years old). They also reported that farmers were the major population, accounting for 87.91% of infected cases. In view of this, our study population seemed to represent the original population very well.

The two frequently used methods to analyse longitudinal data were GEE and random efficient models [19, 20]. We focused on the population average level rather than the difference between patients. The main advantage of GEE resided in the unbiased estimation of population-averaged regression coefficients; thus, we used GEE to analyse the 4-year repeated measured data. The repeated measurements within one individual were correlated with GEE, and the relationship between the variables of the models at different time-points was analysed simultaneously. Articles using GEE for estimation of antibody have been previously published. For example, a vaccine study about Japanese encephalitis predicted the persistence of antibodies after booster vaccination [8]. GEE was a commonly used approach for analysis of follow-up data. To validate our estimation, it would be necessary to keep on measuring neutralising antibody titre for more years.

SFTSV did not form clear plaques on Vero cells [1]. Thus, neutralisation assays of the virus required antibodies and cell staining for detection [21]. These assays were time-consuming and expensive. The problem was addressed by virus passage. We inoculated Vero cells with SFTSV, which were passaged until plaques were clearly visible. In one recent study of a neutralisation assay [22], clear plaques were made in inoculated Vero cells by using a highly passaged SFTSV strain. Furthermore, they performed sequence analysis to determine the characteristics of the strain. Their results suggested that a single amino acid mutation within the viral glycoprotein conferred the ability to make clear plaques to SFTSV.

Two of the patients had higher titres in the fourth year than the first year were excluded as ‘outliers’ in our analysis. When we found patients’ titre were higher at the later follow-up, we tested those samples again to check the correctness. Re-test results showed titre levels were really higher in the 4th year than the 1st year. Though the titre levels were correct, we still decided to exclude the two samples in the sight of immunology. Titres of anti-virus antibodies will arise after infection and tend to be stable in a few weeks and then decline with time. Specific antibodies increase generally when antigens enter the body again, such as secondary infection and vaccination. Our samples were collected in a county named Yiyuan. Previously, our team found that approximately 1% healthy persons were seropositive for SFTSV in Yiyuan County, which indicated Yiyuan was a SFTSV-hit area [23]. Moreover, our samples were from farmers who lived in hilly areas of Yiyuan and engaged in agriculture activities. That meant they were at high risk of becoming infected by this disease. So it might be secondary infection that made the two patients’ titres became higher. Besides, we did a sensitivity analysis including the two patients in our analysis. The predicted persistence of antibodies changed to 11 years, two years more than 9 years when excluding the data. The QIC of predicted model became higher. Based on these reasons we kept the two patients excluded in our analysis.

In our study, we found that GMT was higher in females than in males. The predicted persistence of neutralising antibodies in females was 2 years longer than in males. The result indicated that the immune system response in females was stronger against SFTSV. Males experienced a greater severity and prevalence of bacterial, viral, fungal and parasitic infections than females, who also exhibited a more robust response to antigenic challenges such as infection and vaccination [24, 25]. A study showed that females had, on average, 1.7 times the frequency of self-specific T cells than males [26]. The mechanism behind it had remained a mystery, but one study identified a handful of genes that are apparently regulated by testosterone and are thought to be a key part of the response mechanism. The higher the testosterone levels of a participant are, the lower the immunological reaction to infection or vaccination [27].

In this study, we only recruited 25 patients. The small number is a limitation. It was not easy to recruit a lot of participants in a short period. First, SFTS patients who could be included in our study must be laboratory-confirmed by RT-PCR, a golden diagnostic method. Second, SFTSV was an infectious disease and many patients wanted to be treated privately to avoid affecting their social life. This mind would affect patients’ choice to take part in scientific research though we had provided strict privacy protection.

In conclusion, based on 4 years of follow-up data and mathematical prediction, the protection conferred by neutralising antibodies against SFTSV could last as long as 9 years. The durations of protection were different for different initial titres. The immune system response was stronger against SFTSV in females than in males. The persistence of neutralising antibodies in females was also longer than in males. Insight into the characteristics of neutralising antibodies will allow for better assessment of future SFTSV vaccination. Based on the predicted persistence of neutralising antibodies against SFTSV, our results suggested that booster vaccinations of future vaccines might be scheduled no later than 9 years after the first booster dose.
